# Photoperiodic regulation of Wnts in spermatogenesis of Brandt’s vole (*Lasiopodomys brandtii*)

**DOI:** 10.1186/s12983-026-00596-4

**Published:** 2026-01-21

**Authors:** Lijuan Zhao, Jiajia Shi, Yan Liu, Lewen Wang, Junping Zhao, Yuhua Shi, Hong Sun, Dawei Wang, Zhenlong Wang

**Affiliations:** 1 School of Life Sciences, Zhengzhou University, Zhengzhou, 450001 Henan China; 2 Zhengzhou Key Laboratory of Sport Nutrition and Health, School of Kinesiology and Physical Education, Zhengzhou University, Zhengzhou, 450001 Henan China; 3 Western Agricultural Research Center , Chinese Academy of Agriculture Science, Changji, 831100 China; 4 State Key Laboratory for Biology of Plant Diseases and Insect Pests, Institute of Plant Protection , Chinese Academy of Agricultural Sciences, Beijing, 100193 China

**Keywords:** Seasonal breeding, Photoperiod, Wnt, Testicular development, Spermatogenesis

## Abstract

Photoperiod regulates spermatogenesis in seasonal breeders, yet its molecular mechanisms remain unclear. The Wnt signaling pathway is critical for spermatogenesis in non-seasonal breeders, but its role in photoperiodic spermatogenesis in seasonal breeders is unexplored. To investigate this, we exposed male Brandt’s voles (*Lasiopodomys brandtii*), a strict seasonal breeder, to long-photoperiod (LP, 16L:8D) and short-photoperiod (SP, 8L:16D) from the embryonic stage. Testis volume was markedly larger in LP than in SP from 4 to 10 weeks after birth. RNA-seq analysis of 151 Wnt pathway genes revealed differential expression, with *Wnt5a*, *Wnt6*, *Wnt7a,* and *Wnt9a* downregulated and *Wnt7a* upregulated in LP compared to SP. RT-qPCR and Western blotting confirmed these patterns. Immunofluorescence analysis showed that WNT7A and WNT9A were localized to pachytene and diplotene spermatocytes at 4 weeks and to elongating spermatid heads at 10 weeks. WNT6 and WNT5A appeared as perinuclear foci in early spermatocytes at 4 weeks, with WNT6 subsequently shifting to spermatid nuclei at 10 weeks. These findings indicate that prenatal and postnatal photoperiodic exposure, potentially mediated through both maternal and direct pathways, is associated with distinct spatiotemporal expression patterns of WNT5A, WNT6, WNT7A, and WNT9A, suggesting their potential involvement in seasonal reproduction.

**Supplementary Information:**

The online version contains supplementary material available at 10.1186/s12983-026-00596-4.

## Introduction

Seasonal breeding is a naturally occurring phenomenon in which the reproductive system of adult mammals is activated and deactivated, thereby increasing the likelihood of producing viable chances of successful offspring [1, 2]. This strategy is a widespread among mammals inhabiting environments characterized by substantial seasonal fluctuations outside of stable or weakly fluctuating, rather than stable or weakly variable habitats, such as those occupied by domestic, synanthropic, tropical, coastal, or marine mammals, with predictable fluctuations. Photoperiod, the daily duration of light exposure, is a key regulator of seasonal breeding [3, 4]. In seasonal breeders, light signals detected by retinal photoreceptors trigger hormonal changes that modulate testicular function, promoting spermatogenesis during breeding seasons and inducing regression during non-breeding seasons [5–7]. This results in enlarged testes with active spermatogenesis in long photoperiods (LP) and reduced testis volume with germ cell apoptosis in short photoperiods (SP), as observed in hamsters, deer mice, brown bears, moles, and armadillos [1, 8–13]. Brandt’s vole (*Lasiopodomys brandtii*), a rodent native to the Mongolian plateau, is an ideal model for photoperiodic studies due to its strict seasonal breeding, with significantly larger testis volume in LP compared to SP [14, 15].

Spermatogenesis, the process of sperm production in the testes, is tightly regulated by photoperiod in seasonal breeders [16, 17]. In Syrian hamsters, SP inhibits the production of type A undifferentiated spermatogonia and preleptotene cells [18], while in Plateau poke (*Ochotona curzoniae*), SP leads to their accumulation [19]. In Brandt’s vole, LP enhances spermatogenesis, with increased spermatocytes at 4 weeks after birth and more mature sperm at 10 weeks after birth [20]. Several signal pathways participate in these photoperiodic effects. For instance, SP upregulates Bax/Bcl2 expression in the testis and LC3II/LC3I in the epididymis, accompanied by mitochondrial cristae abnormalities and degradation in testicular reproductive cells in *Cricetulus barabensis* [21]. In Brandt’s vole (*Lasiopodomys brandtii*), the PINK1-mediated mitophagy pathway is also activated in spermatocytes under short photoperiod [20]. Testicular germ cell apoptosis is also found in Syrian hamsters, Iberian mole, and white-footed mice treated with short light [18, 22, 23]. Recent studies also report that retrotransposons participate in photoperiodic spermatogenesis in Brandt’s vole by co-transcription with flagellar genes [24]. However, the involvement of other signaling pathways in photoperiodic spermatogenesis remains unexplored.

The Wnt signaling pathway is critical for testicular spermatogenesis in non-seasonal breeders such as mice [25]. Canonical Wnt signaling stabilizes β-catenin to promote germ cell proliferation, while its disruption causes spermatogenic arrest and apoptosis [26–28]. Specific Wnt genes demonstrate compartmentalized functions: *Wnt3a* is expressed in round and elongating spermatids in male mice reproductive tissues [29]; *Wnt3a/5a/10b* promote spermatogonia stem cell proliferation [30]; Overexpressed *Wnt4* induces germ cell apoptosis [31]; *Wnt7a* knockout leads to infertility with reduced sperm count [32]; *Wnt6* mediates the proliferation of undifferentiated spermatogonia in adult mice [29]. Despite these insights, the role of the Wnt signaling pathway in seasonal breeders is unknown.

To address this gap, we hypothesized that the Wnt signaling pathway is involved in photoperiodic spermatogenesis in Brandt’s voles. We exposed male Brandt’s voles to LP (16 h light / 8 h dark) and SP (8 h light / 16 h dark) from the embryonic stage, analyzed testicular morphology and function weekly from 4 to 10 weeks after birth, and performed transcriptomic profiling of 151 Wnt pathway genes. Expression and localization of *Wnt5a*, *Wnt6*, *Wnt7a*, and *Wnt9a* were validated using RT-qPCR, Western blotting, and immunofluorescence. Our results characterize the expression patterns of the Wnt signaling pathway during photoperiodic spermatogenesis.

## Materials and methods

### Animals and photoperiod treatments

Brandt’s voles (*Lasiopodomys brandtii*), aged 8–10 weeks (reproductive maturity) and weighing 30–40 g, were sourced from the Institute of Plant Protection, Chinese Academy of Agricultural Sciences (Beijing, China). Animals were housed in polycarbonate cages (37 × 26 × 17 cm^3^) with one male and two females at 23 ± 1 ℃ under a 14 h light/10 h dark cycle (200 Lux). Upon detecting vaginal plugs, pregnant females were randomly assigned to either a long photoperiod group (LP, 16 h light/ 8 h dark) (07:00–23:00) or a short photoperiod (SP, 8 h light/ 16 h dark) (07:00–15:00). Newborn males were weaned at 3 weeks and housed individually under their respective photoperiods until 10 weeks. Food and water were provided ad libitum. All procedures complied with National Institutes of Health guidelines and were approved by the Institutional Animal Care and Use Committee of Zhengzhou University (No ZZUIRB GZR 2023–0084).

### Testis volume measurement

To assess testicular development, testis volume was measured in at least 12 male voles per group. Measurements were taken weekly at 09:00 every Monday, from 4 to 10 weeks after birth. At 4 and 10 weeks, 12 male voles per group were anesthetized with ether, weighed, and euthanized by decapitation. Bilateral testis weight and volume were recorded, with testis volume calculated as (4/3) π × (R1 × R2^2^), where R1 is half the long axis, and R2 is half the short axis.

### RNA extraction and sequencing

To investigate photoperiod-regulated gene expression, testes were collected from 24 male voles under long and short photoperiods at 4 and 10 weeks. One testis was fixed and embedded in paraffin for subsequent immunofluorescence analyses. The contralateral testis from the same animal was used for total RNA extraction. Total RNA was extracted using Trizol reagent (Thermo Fisher Scientific, Waltham, USA) [33] and treated with RNase-free DNase I (Thermo Fisher Scientific, Waltham, USA) at 37℃ for 30 min [33]. RNA quality and quantity were assessed using a NanoDrop spectrophotometer (260/280 nm). cDNA libraries were constructed and sequenced on the Illumina HiSeq 4000 platform (Novogene, Beijing, China).

### Quality control of RNA-seq results

To ensure data reliability, raw RNA-seq reads were preprocessed to remove adaptor sequences and low-quality reads (defined as reads containing N > 10% bases or > 50% bases with Q-value ≤ 5), yielding clean reads with a Q30 ratio > 92% and GC content of 49% — 53% (Supplementary Table 1). Reads were aligned to the Brandt’s vole reference genome (PRJNA523083) using HISAT2 (v2.2.1) [34], achieving a mapping ratio of over 91.68% (Supplementary Table 1). Principal component analysis (PCA) confirmed clustering of biological replicates at 4 and 10 weeks (Supplementary Fig. 1), with no significant differences in gene expression within groups. Based on the PCA results, we excluded two outlier samples from the data analysis pipeline at 4 weeks after birth in LP and SP, respectively.

### Wnt pathway gene expression analysis

To examine Wnt signaling, expression data for 151 Wnt pathway genes, retrieved from the KEGG database using keggGet in R Studio (Supplementary Table 2), were extracted from the RNA-seq dataset. A clustered heatmap of transcripts per million (TPM) values was generated using the R package pheatmap (https://cran.rproject.org/web/packages/pheatmap/index.html). Differentially expressed genes (DEGs) were identified with DESeq2 (|log_2_FoldChange|≥ 1, adjusted *P* < 0.05) in RStudio [35]. Volcano plots and Venn diagrams of DEGs at 4 and 10 weeks were created using DESeq2 and TBtools, respectively [36]. Protein–protein interactions were analyzed with STRING (https://string-db.org/) [37], and visualized in Cytoscape [38].

### Quantitative real-time polymerase chain reaction (RT-qPCR)

To validate RNA-seq findings, RT-qPCR was performed using the same total RNA extracts from the same cohort of animals used for RNA-seq. RNA (1 μg) was reverse-transcribed into cDNA using HiScript III 1st Strand cDNA Synthesis Kit (Vazyme, R312-01, Nanjing, China). For qPCR, equal amounts of cDNA from six individuals per group were used. RT-qPCR was performed on a Light Cycler 480 (Roche, U.S.A.) with SYBR Premix EX Taq II (TaKaRa). Primers for β-actin (reference gene) and Wnt pathway genes are listed in Table 1. Amplification efficiency was 0.9—1.0, with no amplification in non-template controls. Expression fold changes were calculated using the 2^−∆∆Ct^ method. Pearson’s correlation analysis, performed in GraphPad Prism v9.0.0, assessed concordance between RT-qPCR and transcriptome data [39].

**Table 1:** RT-qPCR primers for key genes of the Wnt signal pathway

Gene	Forward (5’-3’)	Reverse (3’-5’)
Wnt5a	CATAGCCGGAACCTACGTGA	TCTTTGATGCCTGTCTTCG
Wnt6	GTTCCAGTTCCGTTTCCGAC	GGCTGTCTCTCGAATGTCCT
Wnt7a	CAATAAGACAGCCCCTCAAGC	TGTACATCTCGGTGCGCTCA
Wnt9a	AGTACAGCAGCAAGTTTGTCA	CTACTCCGGCTTTTATCACCT
β-actin	ACATCCGTAAAGACCTCTATGC	TACTCCTGCTTGCTGATCCAC
Axin2	TACCTCCCCACCTTGAACGAA	CATAGCCGGAACCTACGTGA
Csnkl1e	ACATCATTGACTTCGGCTTGG	TCAGGTTCTTGTTTTCCCGGTA
Fzd7	CCATGAGCCCCGACTTCACA	ACCAGATCCAGAAGCCGGTA
Fzd8	GAAACCAGAGCCTCGACAACC	GACACGAAACCAGCCAACAGA
Fzd10	TTCCTTCATCCTGTCCGGCTT	CCAGCATCTTCCAGTAGTCCA
Jun	GACATGGAGTCTCAGGAGCG	CTCCGAGTTTTGCGCTTTCA
Lef1	CCTCATCCAGCTATTGTAACAC	TCCTGCTCCTTTCTCTGTTCG
Lrp6	CCAGAGCTATTGCCTTAGACCC	ATCTGCCCAATAAAGCTTCCG
Senp2	ATCAGCCACGTAGAGTCCT	TCCGAAACCATCTCTGTTACACC
Tcf7L2	AAAGTGCAGCCATTAACCAGA	ATAGTTATCCCGCGCAGACCA
Wif1	AGCCATTCCTGTCAATATCCAC	CTGCCATGATGCCTTTATCCAG

### Western blot analysis

To quantify Wnt protein expression, testis tissues were homogenized in lysis buffer (10 mM Tris–HCl, 1 mM EDTA, 2.5% SDS, with Complete™ protease inhibitor cocktail). Protein (50 μg) was separated by SDS-PAGE and transferred to 0.45 μm PVDF membranes (Bio-Rad, Hercules, CA, USA; Cat. No. 162–0177). Membranes were blocked with 5% non-fat milk in TBST (0.14 M NaCl, 2.7 mM KCl, 25 mM Tris, 0.1% Tween-20, pH 7.4) for 2 h at 4 °C, then incubated with primary antibodies for 2 h at room temperature: WNT7A (1:3000; Signalway antibody, College Park, MD, USA; Cat. No. 38653), WNT6 (1:2000; Abcam Cambridge, UK; Cat. No. ab150588), WNT9A (1:1000; Signalway antibody, College Park, MD, USA; Cat. No. 335983), WNT5A (1:1000; BD Biosciences, Franklin Lakes, NJ, USA; Cat. No. 610154). After washing with TBST, membranes were incubated with HRP-conjugated anti-rabbit IgG (1:5,000; Vector Laboratories, Burlingame, CA, USA; Cat. No. PI-1000) for 1 h at room temperature. Chemiluminescent detection was performed using SuperSignal™ West Pico PLUS substrate (Thermo Fisher Scientific, Waltham, MA, USA; Cat. No. 34580) with Hyperfilm™ ECL (GE Healthcare, Chicago, IL, USA; Cat. No. 28906839). β-Tubulin (1:3,000; Abcam; Cambridge, UK; Cat. No. ab8229) served as the loading control. Band intensities were quantified using ImageJ software [40].

### Immunofluorescence

To examine Wnt protein localization, fresh testes were fixed with 4% paraformaldehyde for 24 h, dehydrated through a graded ethanol series (75% for 4 h, 85% for 2 h, 90% for 2 h, 95% for 1 h, 100% ethanol I for 30 min, 100% ethanol II for 30 min, ethanol–benzene for 5–10 min, xylene I and II for 5–10 min each), and embedded in paraffin at 65 ℃ (three 1 h immersions). Sections (5 μm) were deparaffinized, rehydrated, and subjected to epitope retrieval using EDTA (pH 9.0) at 90 ℃ for 20 min. Section was blocked with 1% goat serum in PBS for 1 h and incubated overnight at 4℃ with primary antibodies diluted in PBS: WNT7A (1:300), WNT6 (1:200), WNT9A (1:500), WNT5A (1:400). After washing, sections were incubated with Alexa Fluor® 488 secondary antibodies (1:500, Abcam, Cat. No. ab150169) for 1 h at room temperature. Nuclei were counterstained with 500 ng/mL 4,6-diamidino-2-phenylindole (DAPI) in PBS. Images were captured using the Vectra Automated Quantitative Pathology Imaging System (PerkinElmer, Waltham, MA, USA) at 350 nm (DAPI) and 488 nm (WNTs) with uniform exposure settings.

### Statistical analyses

Analysis of testicular developmental phenotype under different photoperiods was performed using repeated-measures analysis. Two-way ANOVA followed by Bonferroni correction for multiple comparisons was used to assess the effects of photoperiod and time on gene expression levels. Independent sample t-tests were used for between-group comparisons of protein levels within the same week, as samples from 4 and 10 weeks were processed on separate gels/membranes. Normality and homogeneity of variance were tested prior to analysis; non-parametric tests were applied where assumptions were not met. All analyses were conducted using SPSS version 22.0 (IBM, Armonk, NY, USA). Data are presented as mean ± SEM, with statistical significance set at *P < 0.05, **P < 0.01, and ***P < 0.001. Graphs were generated using GraphPad Prism 8.0.2 (GraphPad Software, San Diego, CA, USA).

## Results

### Photoperiod effects on testicular development

**Fig. 1:**
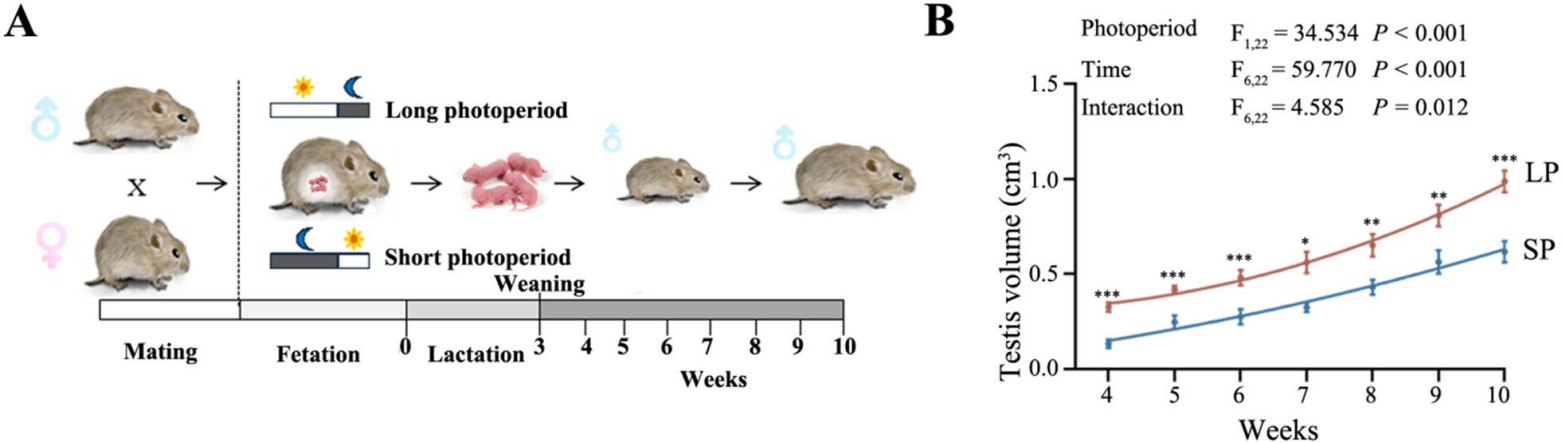
Effect of different photoperiods on the postnatal testicular development in male Brandt’s vole. **A** The flowchart of the experimental design for this study. **B** Testis volume changes over time under LP and SP. LP, long photoperiod. SP, short photoperiod

To assess the impact of photoperiod on testicular development, we exposed Brandt’s voles to long (LP, 16 h light/ 8 h dark) or short (SP, 8 h light/16 h dark) photoperiods from the embryonic stage, and monitored testis volume weekly from 4 to 10 weeks (Fig. 1A). Testis volume increased over time in both groups, but LP exhibited significantly larger testes. At 4 weeks, testis volume was 0.33 ± 0.02 cm^3^ in LP and 0.13 ± 0.02 cm^3^ in SP. By 5 weeks, volume increased by 28.32% in LP and 83.75% in SP. At 10 weeks, LP reached 0.99 ± 0.06 cm^3^ (22.32% growth rate), while SP testes reached 0.62 ± 0.06 cm^3^ (9.58% growth rate) with a marked difference between groups (Fig. 1B). These findings indicate that long photoperiods promote testicular development in Brandt’s voles.

### Wnt signaling pathway analysis in testis transcriptome

**Fig. 2:**
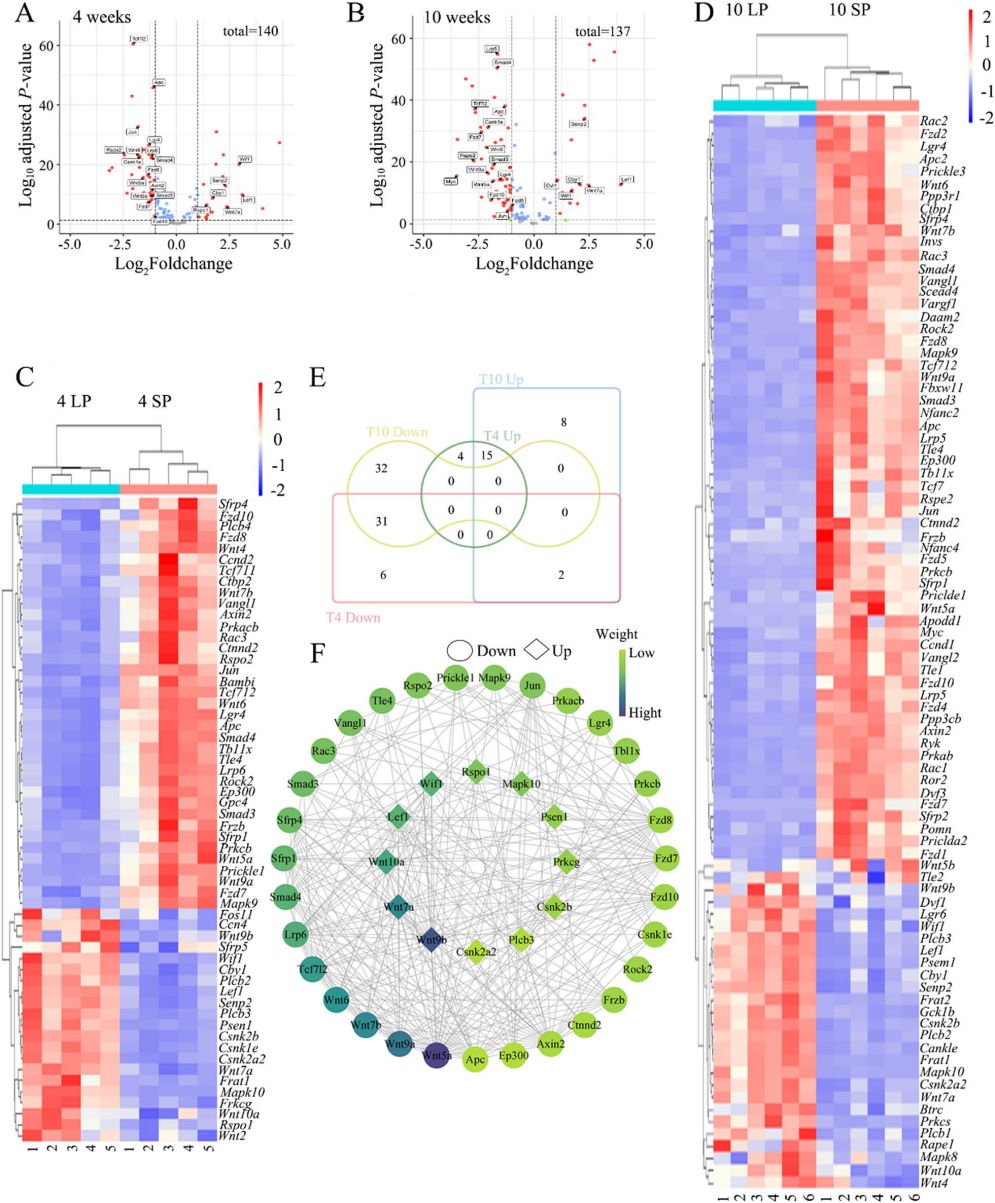
Transcriptomic analysis of the Wnt signaling pathway in Brandt’s vole testes. Volcano plot of differentially expressed genes of the Wnt signaling pathway in the testis at 4 **A** and 10 **B** weeks. Red circles represent DEGs, with a threshold of |log_2_FoldChange|≥ 1 and adjusted *P* value < 0.05. Heatmap of DEGs of the Wnt signaling pathway in the testis at 4 **C** and 10 **D** weeks. **E** Venn Diagram of common DEGs. **F** Protein–protein interaction network of common DEGs, with darker color indicating stronger interactions. The circles and squares indicate that the gene encoding each protein is downregulated and upregulated in the LP compared to the SP group, respectively. LP, long photoperiod. SP, short photoperiod

To elucidate the role of the Wnt signal pathway in photoperiodic testicular development, we conducted RNA-seq on testis samples from 22 male Brandt's voles exposed to LP and SP at 4 and 10 weeks. Then, we analyzed the expression levels of 151 Wnt pathway genes in LP and SP using the testes transcriptome. At 4 weeks, 58 DEGs were detected, with 39 downregulated and 19 upregulated in LP compared to SP (Fig. 2A). At 10 weeks, 88 DEGs were identified, including 63 downregulated and 25 upregulated genes (Fig. 2B). Heatmaps revealed four distinct clusters, indicating significant expression differences between LP and SP (Fig. 2C, D). Among the 46 common DEGs at both time points, 15 were upregulated and 31 downregulated in LP (Fig. 2E). Among these, 15 were randomly selected for RT-qPCR. The correlation analysis results indicated that the transcriptome sequencing and analysis results were reliable (Supplementary Fig. 2). Protein–protein interaction (PPI) analysis using STRING and visualized in Cytoscape highlighted WNT5A, WNT6, WNT7A, and WNT9A as key nodes with extensive interactions (Fig. 2F). These findings suggest that the Wnt signaling is dynamically modulated by photoperiod and coincides with the photoperiodic effects on testicular development in Brandt’s voles.

### Expression level of Wnts in the testes of Brandt’s vole

**Fig. 3:**
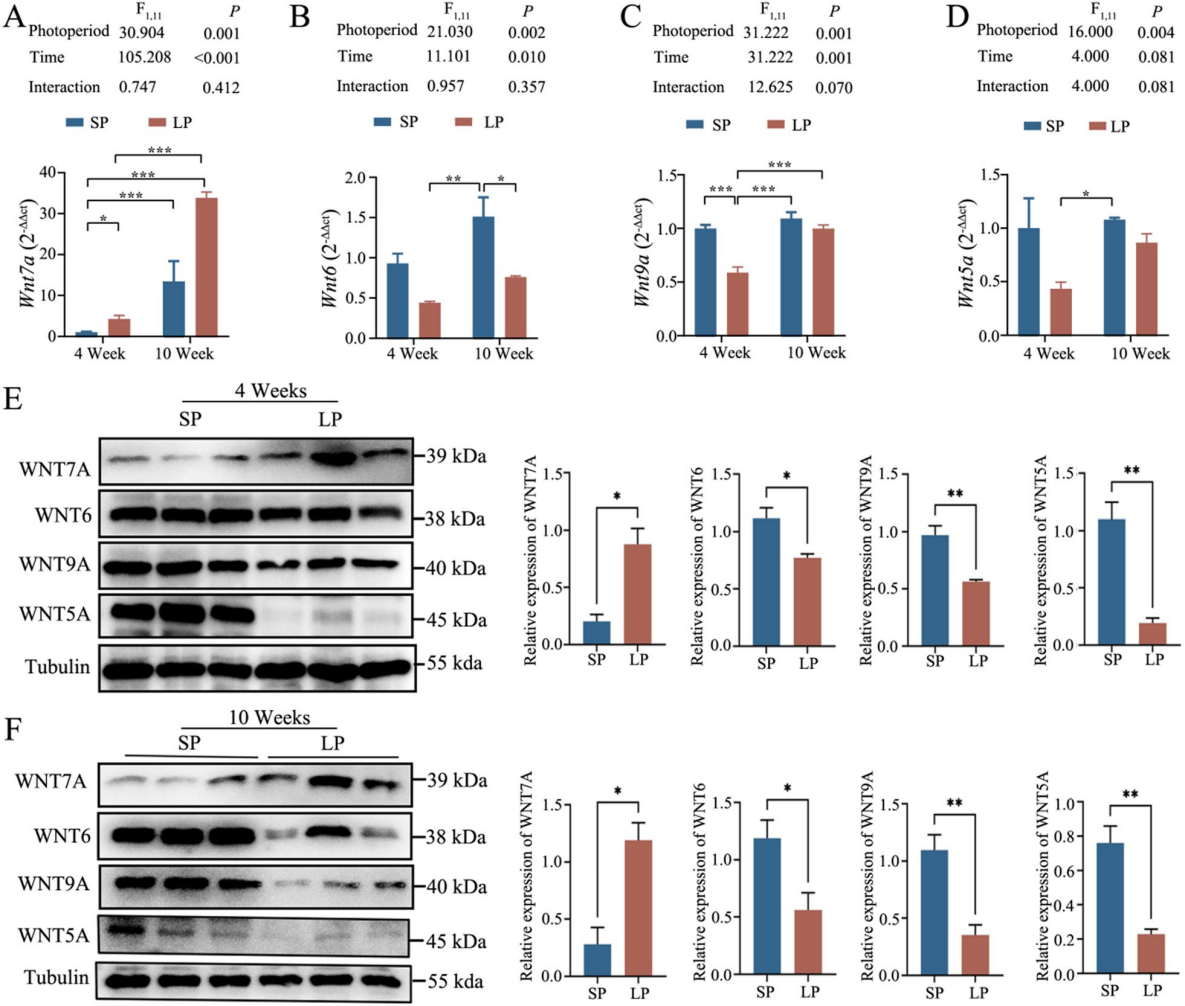
Expression of the Wnt pathway genes under different photoperiods. RT-qPCR results for *Wnt7a* **A**, *Wnt6* **B**, *Wnt9a* **C**, and *Wnt5a* **D** at 4 and 10 weeks. Western blotting results for WNT7A, WNT6, WNT9A, and WNT5A in 4 **E** and 10 **F** weeks after birth. Data are mean ± SEM (* *P* < 0.05, ** *P* < 0.01, *** *P* < 0.001)

Building on the transcriptomic identification of key Wnt genes, we used RT-qPCR and Western blotting to validate the expression levels of *Wnt5a*, *Wnt9a*, *Wnt7a*, and *Wnt6* under LP and SP. Two-way ANOVA analysis showed that photoperiod and time significantly affected *Wnt7a*, *Wnt9a,* and *Wnt6* expression, but treatment time and their interaction had no effect (Fig. 3A–C). *Wnt5a* expression was significantly influenced by photoperiod, but not time and their interaction (Fig. 3D). Western blotting confirmed these patterns, with WNT5A, WNT9A, and WNT6 levels lower in SP and WNT7A levels higher in LP at both 4 and 10 weeks (Fig. 3E and F), validating the reliability of transcriptomic results. These findings indicate that photoperiod modulates Wnt pathway gene expression during testicular development.

### Localization of Wnt proteins in the testis of Brandt’s vole

**Fig. 4:**
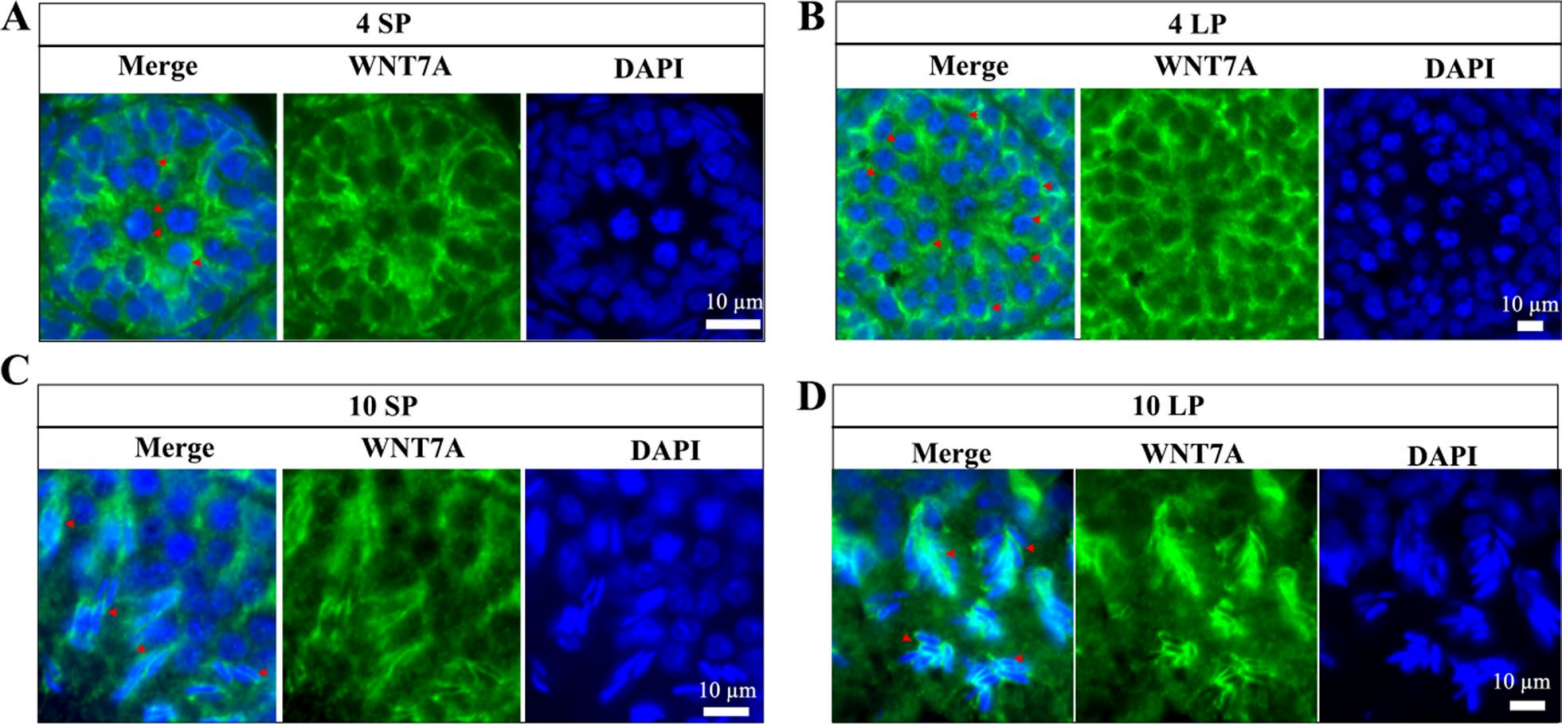
Localization of WNT7A in Brandt’s vole testes. Immunofluorescence results for WNT7A at 4 weeks under SP (A) and LP (B). Immunofluorescence results for WNT7A at 10 weeks under SP (C) and LP (D). Arrows indicate diplotene spermatocytes (A, B) and elongating spermatid heads (C, D)

To further investigate how these key Wnt signaling pathway genes function in testicular development, we utilized immunofluorescence to examine the distribution of WNT7A, WNT5A, WNT9A*,* and WNT6 in testes under LP and SP. At 4 weeks, WNT7A was uniformly distributed in the cytoplasm of spermatogenic cells, particularly in the diplotene spermatocytes in SP and LP (Fig. 4A and B). It was interesting that stronger WNT7A signals were observed at the leptotene-zygotene to pachytene-diplotene interface in LP (Fig. 4B). At 10 weeks, the positive signal of WNT7A mainly appeared at the head of the elongating spermatid (Fig. 4C and D).

**Fig. 5:**
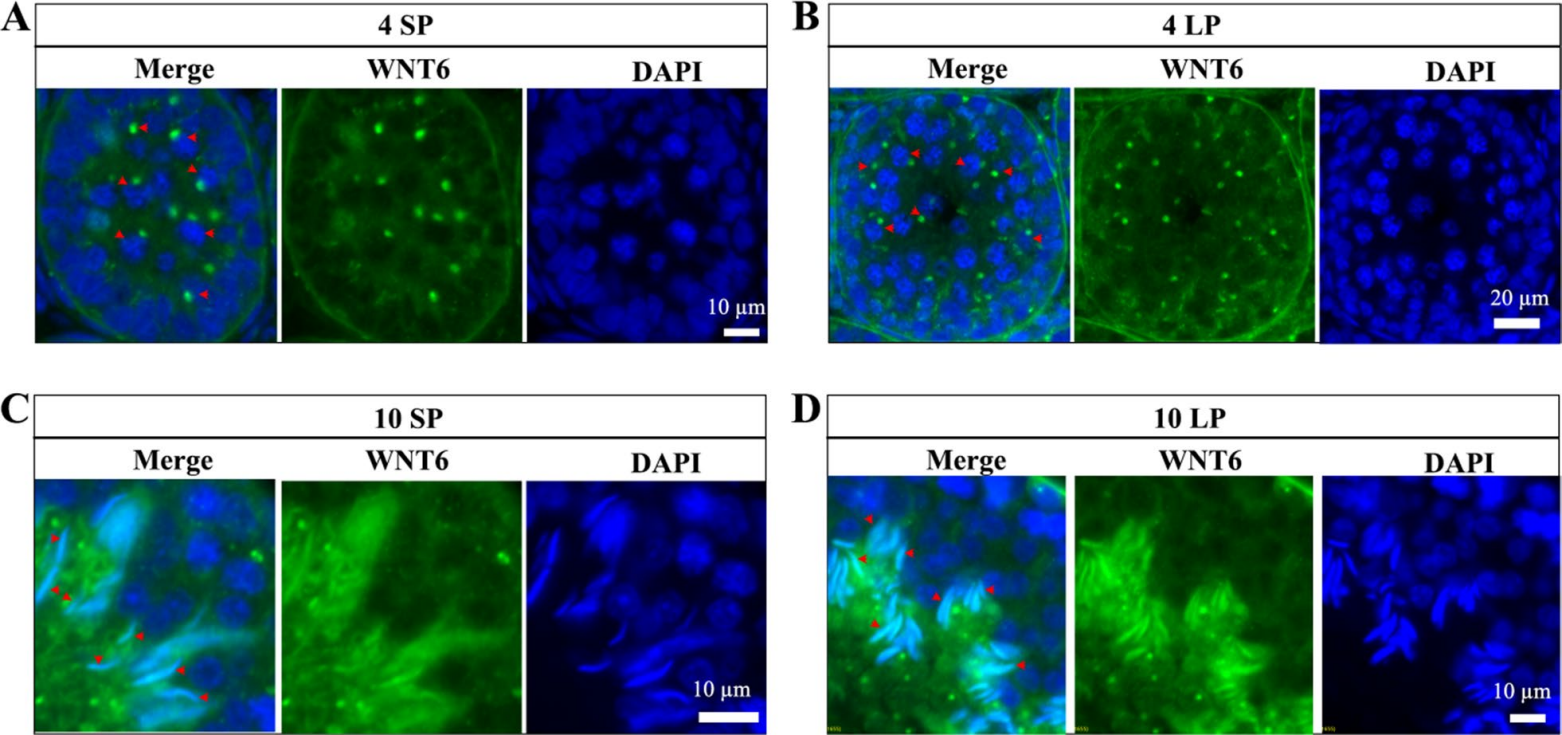
Localization of WNT6 in Brandt’s vole testes. Immunofluorescence results of WNT6 at 4 weeks under SP (A) and LP (B). Immunofluorescence results of WNT6 at 10 weeks under SP (C) and LP (D). Arrows indicate perinuclear foci (A, B) and spermatid nuclei (C, D)

At 4 weeks, WNT6 was observed in the cytoplasmic region of pachytene and diplotene spermatocytes as a single large light spot near the nucleus (Fig. 5A and B). At 10 weeks, the major positive signal was in the nuclei of elongating spermatids in SP and LP (Fig. 5C and D).

**Fig. 6:**
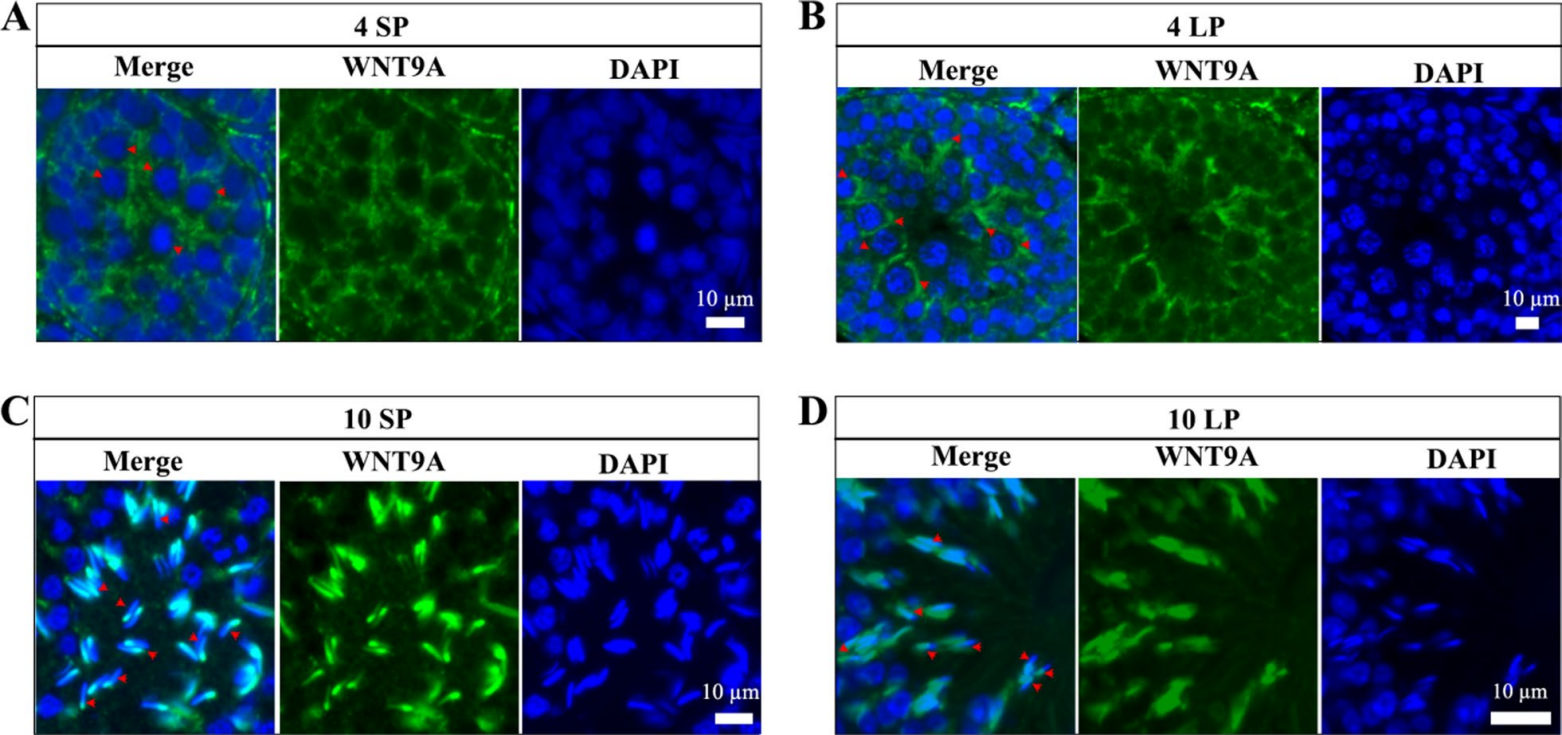
Localization of WNT9A in Brandt’s vole testes. Immunofluorescence results of WNT9A at 4 weeks under SP (A) and LP (B). Immunofluorescence results of WNT9A at 10 weeks under SP (C) and LP (D). Arrows indicate diplotene spermatocytes (A, B) and spermatid heads (C, D)

At 4 weeks, the positive signal of WNT9A appeared in the cytoplasm of pachytene and diplotene spermatocytes, both in SP and LP (Fig. 6A and B). At 10 weeks, the positive signal of WNT9A was also most focused on the head of the elongating spermatid in SP and LP (Fig. 6C and D).

**Fig. 7:**
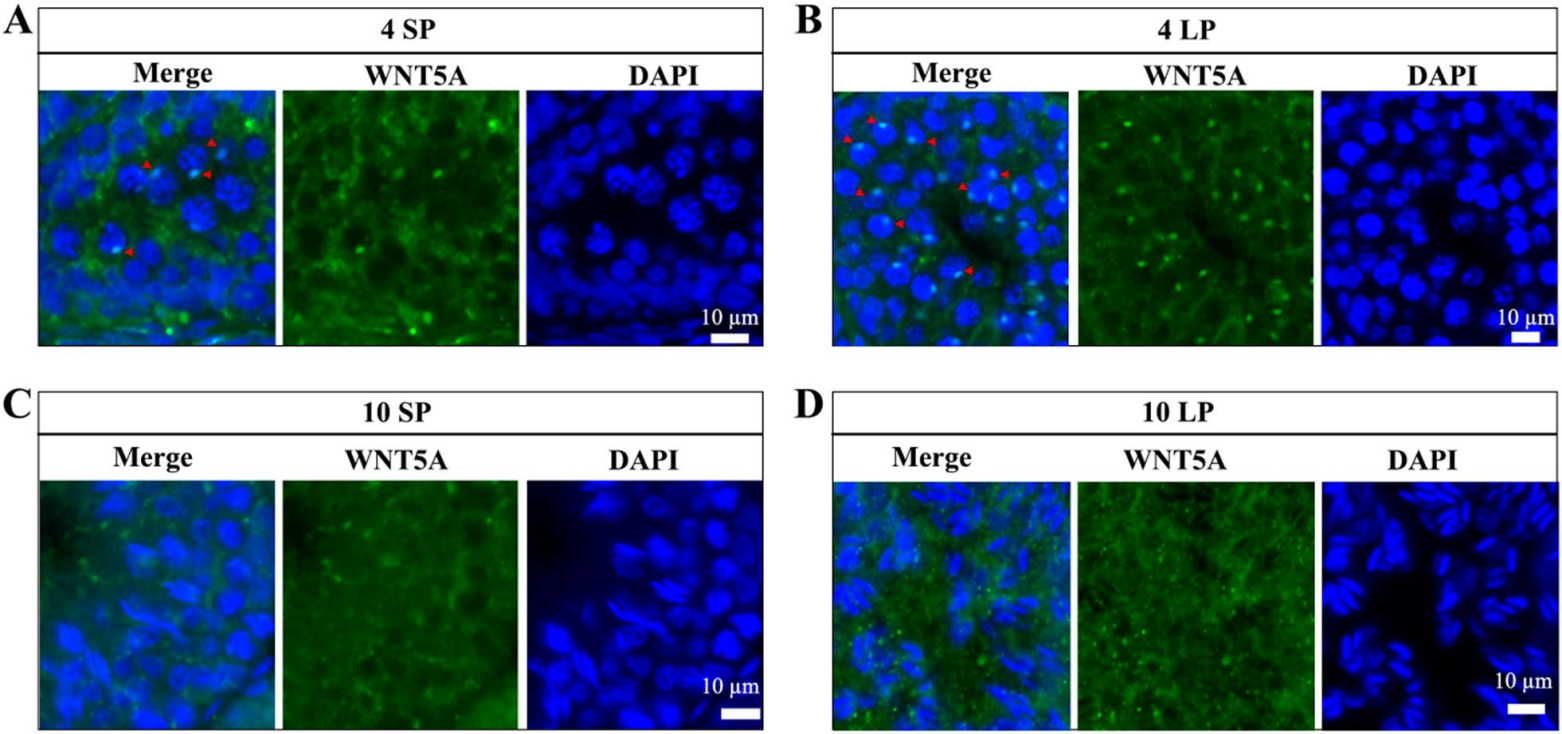
Localization of WNT5A in Brandt’s vole testes. Immunofluorescence results of WNT5A at 4 weeks under SP (A) and LP (B). Immunofluorescence results of WNT5A at 10 weeks under SP (C) and LP (D). Arrows indicate perinuclear foci (A, B)

At 4 weeks, the distribution of WNT5A resembled that of WNT6, localizing to the cytoplasm of the pachytene and diplotene spermatocytes as a single large light spot near the nucleus under both SP and LP (Fig. 7A and B). At 10 weeks, the signal of WNT5A was relatively scattered, and there was no obvious specific positioning signal in the elongating spermatid under SP and LP (Fig. 7C and D).

These distinct localization patterns suggest that the Wnt proteins regulate specific stages of spermatogenesis under photoperiodic control.

## Discussion

In this study, we used different photoperiods to treat male Brandt's voles from the embryonic stage and found that testicular development was promoted in long photoperiods. Then, we analyzed the transcription levels of the Wnt signaling pathway based on the transcriptome of the total testis and found that the key Wnt functions in testicular development, *Wnt5a*, *Wnt6*, *Wnt9a*, and *Wnt7a*. We then performed RT-qPCR, Western blotting, and immunofluorescence to detect the expression level and distribution of *Wnts*. By integrating transcriptomic, molecular, and histological analyses, we demonstrate that photoperiod modulates testicular development of Brandt’s voles is associated with selective changes in Wnt ligand expression. This study provides the first experimental evidence that prenatal and postnatal photoperiodic exposure, potentially mediated through both maternal and direct pathways, programs the spatiotemporal expression of Wnt genes, providing novel mechanisms into seasonal reproduction.

### Photoperiodic regulation of testicular development

Seasonal change in photoperiod is an important environmental factor for rodents, influencing their morphology, physiology, behavior, and reproductive state [41]. Long and short photoperiods, mimicking summer and winter conditions, can be artificially simulated in the laboratory [42]. In this study, we found that the significant differences in testis volume observed at 4 weeks after birth could very likely be influenced by the maternal hormonal environment during gestation and lactation, as the photoperiodic exposure started from the embryonic stage. However, our longitudinal experimental design provides additional compelling evidence for the ongoing, postnatal role of photoperiod. The key finding is that the difference in testicular development between the LP and SP groups was not static but continued to diverge significantly from 4 to 10 weeks after birth. This continuous and divergent growth trajectory strongly suggests that while maternal effects may have established an initial disparity, the postnatal photoperiodic environment actively and directly regulates testicular development in the juvenile and adolescent male voles themselves. This phenomenon is consistent with observations in other species, such as the black-lined hamster and Syrian hamsters [43–45], indicating that the regulation of photoperiod on testicular development is widespread in seasonal breeding animals.

It is important to note that our study, by design, isolated the role of photoperiod under controlled laboratory conditions. Brandt's vole, as a short-lived rodent with a distinct reproductive lifespan, exhibits seasonality in the wild that is shaped by a suite of environmental drivers, including temperature, food availability, and population density [46, 47]. These factors can interact with or override photoperiodic signals to fine-tune reproductive timing. Therefore, photoperiod should be interpreted as a crucial proximal cue that entrains the reproductive axis, upon which other ecological pressures act to determine the ultimate breeding strategy.

### Wnt signaling pathway in the testis of Brandt’s vole

Numerous studies have demonstrated that the Wnt signaling pathway is crucial for spermatogenesis in non-seasonal breeders such as mice [28, 48], but its function in seasonal breeders is underexplored. To investigate this, we first performed transcriptome analysis of Brandt’s vole subjected to different photoperiod treatments. We established a Wnt signaling pathway gene expression matrix and identified dynamic shifts in Wnt pathway genes: compared to SP, LP exhibited 39 downregulated and 19 upregulated DEGs at 4 weeks, and 63 downregulated and 25 upregulated DEGs at 10 weeks. A Venn diagram showed the DEGs with similar expression trends at 4 and 10 weeks after birth, including 15 upregulated DEGs and 31 downregulated DEGs in LP compared to SP. These findings establish that the Wnt pathway is modulated by photoperiod in Brandt’s vole testes, a novel observation in seasonal breeders.

### Wnts in the spermatogenesis of Brandt's voles

Specific Wnt ligands exhibited distinct spatiotemporal patterns during spermatogenesis in mice [29, 32, 49]. Our findings confirm that WNT7A localizes to pachytene and diplotene spermatocytes at 4 weeks, consistent with previous research [29, 50]. However, stronger WNT7A signaling was observed at the interface between leptotene/zygotene spermatocytes and pachytene/diplotene spermatocytes in the LP group. Given that LP accelerates spermatogenesis [20], this spatial enrichment suggests that WNT7A may facilitate meiosis progression from the early to late spermatocyte stage. Similarly, WNT9A localized to pachytene/diplotene spermatocytes at 4 weeks, and exhibited crescent-like expression at elongating spermatid heads near the nucleus at 10 weeks. This shared spatial pattern implies cooperative roles for WNT9A and WNT7A in acrosome formation during spermiogenesis. Sertoli cell-derived WNT6 activates Wnt/β-catenin signaling in undifferentiated spermatogonia, driving proliferation but not differentiation [29]. In Brandt’s vole, WNT6 localized as distinct perinuclear foci in early spermatocytes and later shifted to elongating spermatid nuclei during spermatogenesis, suggesting a novel role in nuclear shaping. WNT5A regulates the self-renewal of spermatogonia stem cells in mice [48]. In our study, WNT5A, similar to WNT6, was localized to the perinuclear region during the early stages of spermatogenesis, but its expression became weak and irregular in the later stages of spermatogenesis. These spatiotemporal patterns differ from previous research, possibly due to methodological disparities (protein-level immunofluorescence vs. RNA in situ hybridization) or species-specific adaptations. Our findings highlight selective Wnt ligand activation under photoperiodic control, distinguishing Brandt’s voles from non-seasonal breeders. Our findings in this obligate seasonal breeder (Brandt’s voles) provide a clear model for understanding the molecular mechanisms of photoperiodism, which may be applicable or represent an extreme case within the wider spectrum of seasonal reproductive strategies in mammals. Furthermore, our transcriptomic data also revealed differential expression of *Wnt7b* and *Wnt9b*, which are primarily known for their functions in embryonic development [51–53]. While these were not the focus of the present study, their altered expression suggests potential, yet uncharacterized, roles in the testicular environment, meriting investigation in future work.

### Limitations and future directions

An important limitation of our study design should be noted. Since the photoperiodic treatments were applied from the embryonic stage, the observed phenotypic and molecular changes in the male offspring could stem from either the direct effect of photoperiod on the developing offspring or indirect effects mediated by the mother's physiological state (e.g., hormonal profiles such as melatonin and corticosterone). Our current setup does not allow us to distinguish between these direct and indirect pathways. Future studies employing cross-fostering or postnatal photoperiod manipulation would be necessary to dissect the precise contribution of prenatal maternal effects.

Our study also has several limitations. First, while we identified correlations between Wnt expression and spermatogenesis in seasonal breeders, the causal relationships remain unproven. Future experiments using Wnt inhibitors or conditional knockouts could validate these roles. Second, our analysis was restricted to the Wnt pathway; future studies should explore its interactions with other signaling pathways, such as Hedgehog and Notch.

## Conclusion

In conclusion, our study revealed the selective regulatory role of the Wnt signaling pathway in the photoperiodic spermatogenesis in Brandt’s voles, particularly in the temporal and spatial expression patterns of different types of spermatogenic cells. By identifying this pathway as a key regulator, our work provides foundational insights into the molecular mechanisms that govern seasonal reproduction in this species. These findings establish a basis for future comparative studies in other seasonal breeders and contribute to a broader understanding of how environmental cues like photoperiod can orchestrate complex reproductive processes.

## Supplementary Information

Additional file1 (DOCX 5544 KB)

Additional file2 (DOCX 2535 KB)

## Data Availability

The genome sequence data of **Lasiopodomys brandtii**: XGi2017 (Brandt’s vole) used in this study have been deposited in the National Center for Biotechnology Information (NCBI) under accession number PRJNA523083. The RNA-Seq raw data of this study have been deposited in the Sequence Read Archive (SRA) database under accession number PRJNA1092792.
