# Supplementary figures and images for: Photoperiodic regulation of Wnts in spermatogenesis of Brandt’s vole (Lasiopodomys brandtii)
Source: Front Zool. 2026 Jan 21;23:5. doi: 10.1186/s12983-026-00596-4 (PMC12905888; doi:10.1186/s12983-026-00596-4)

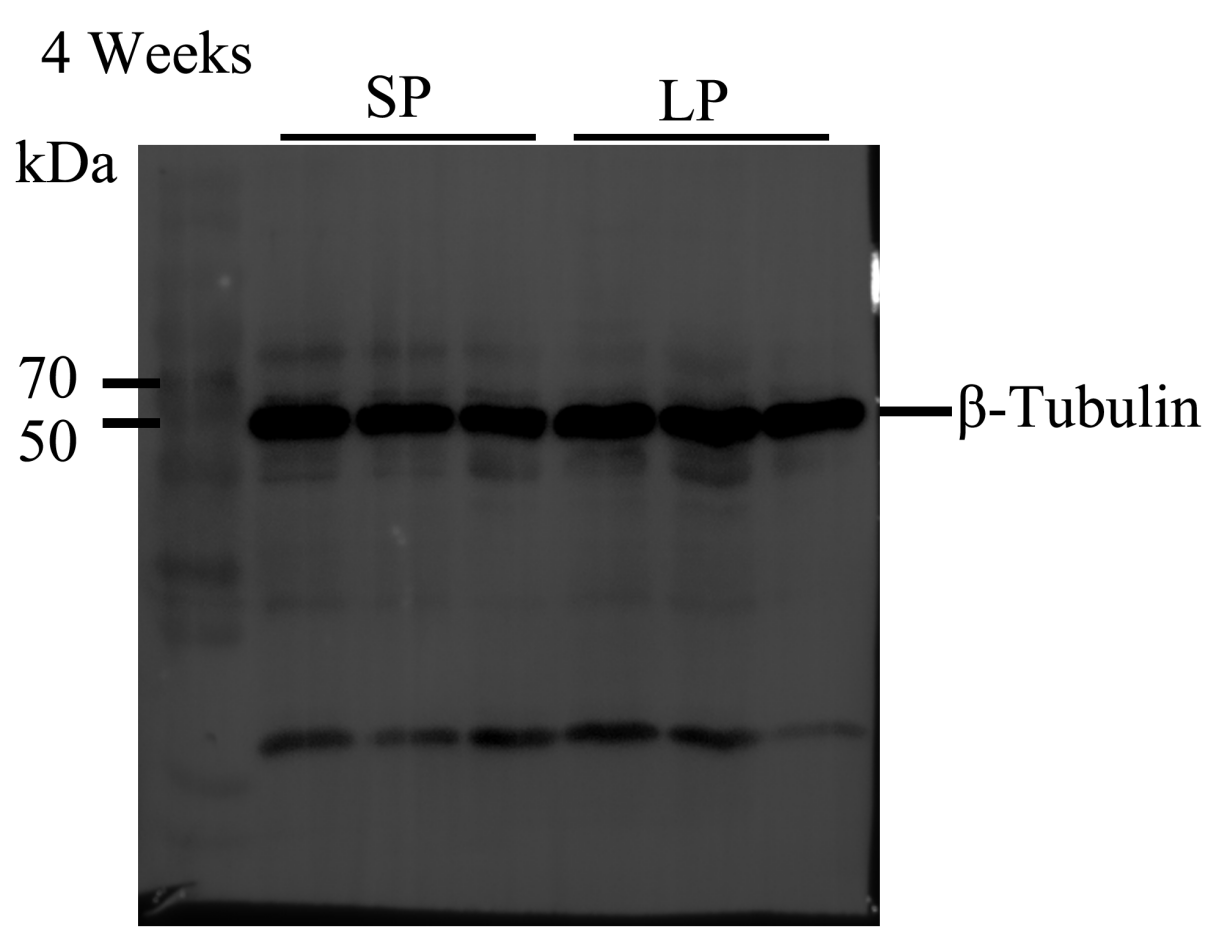

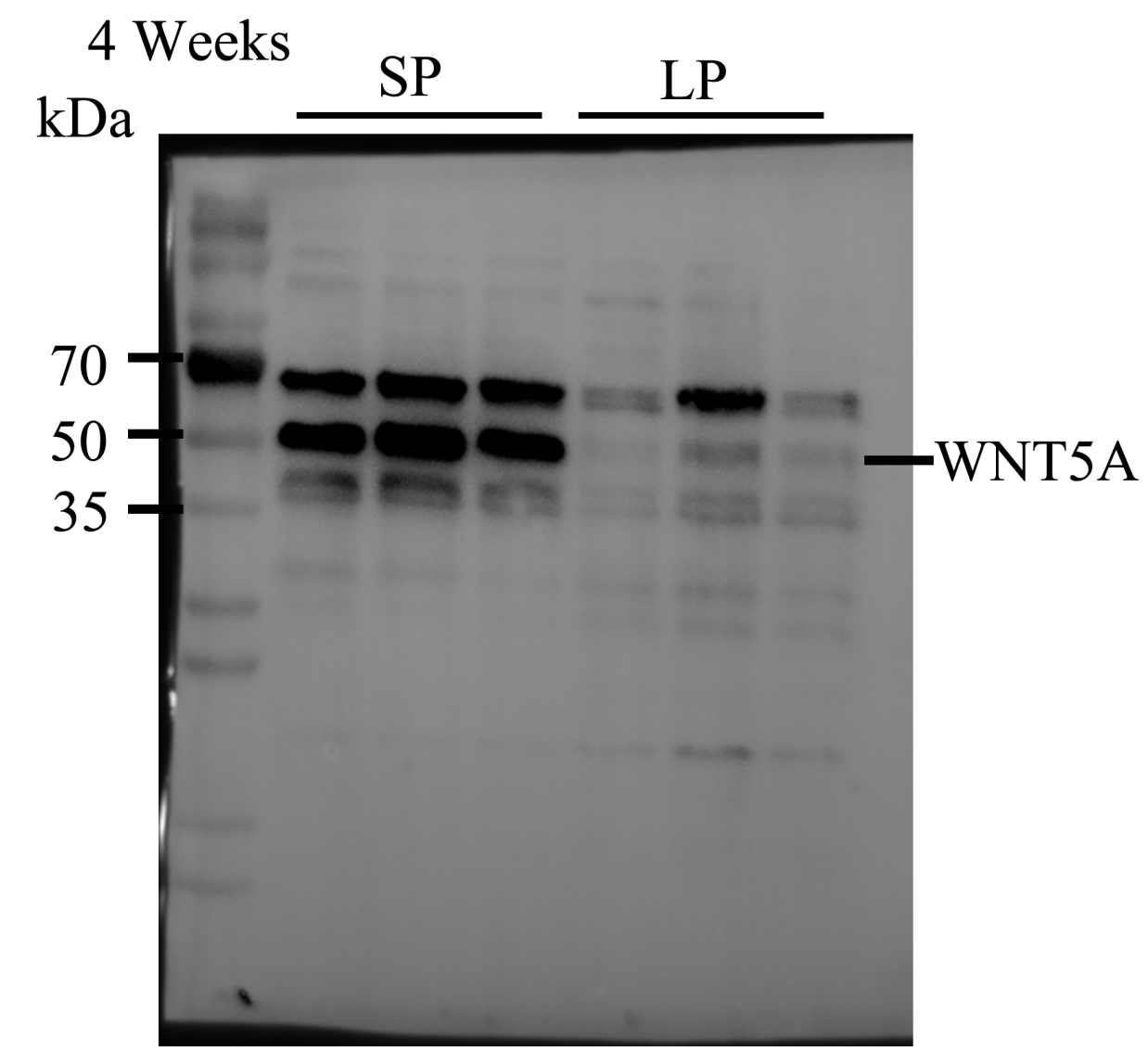

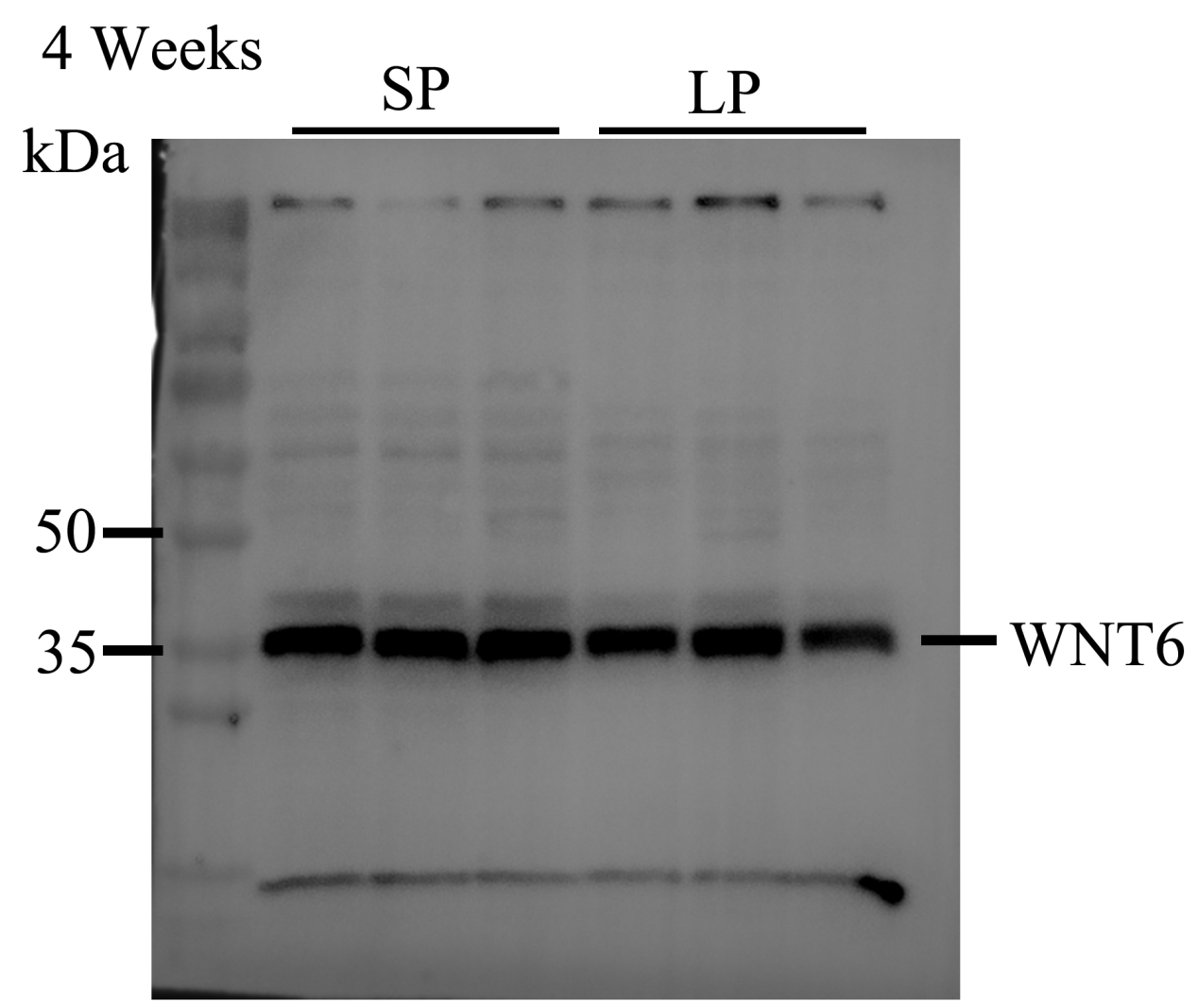

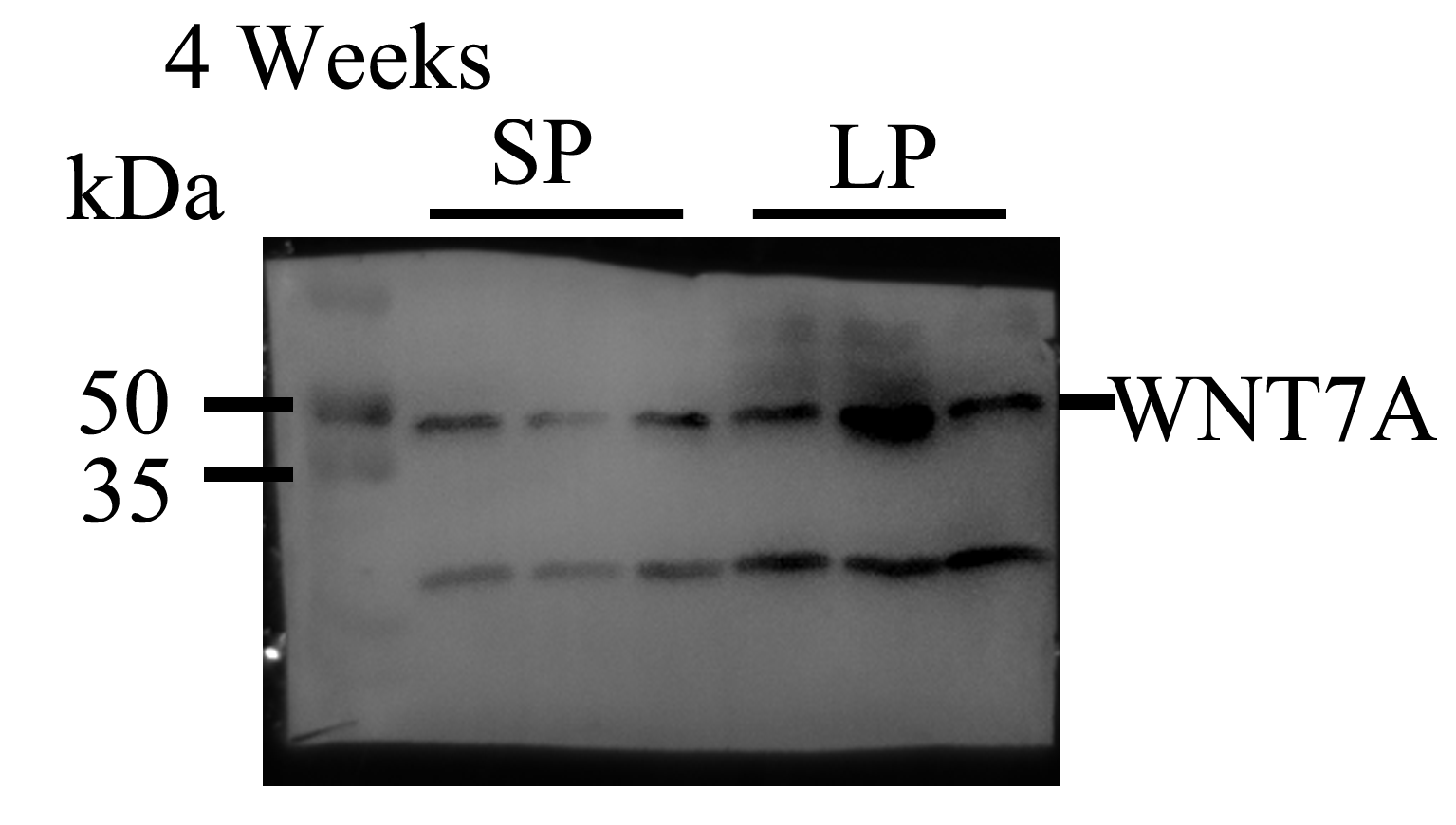

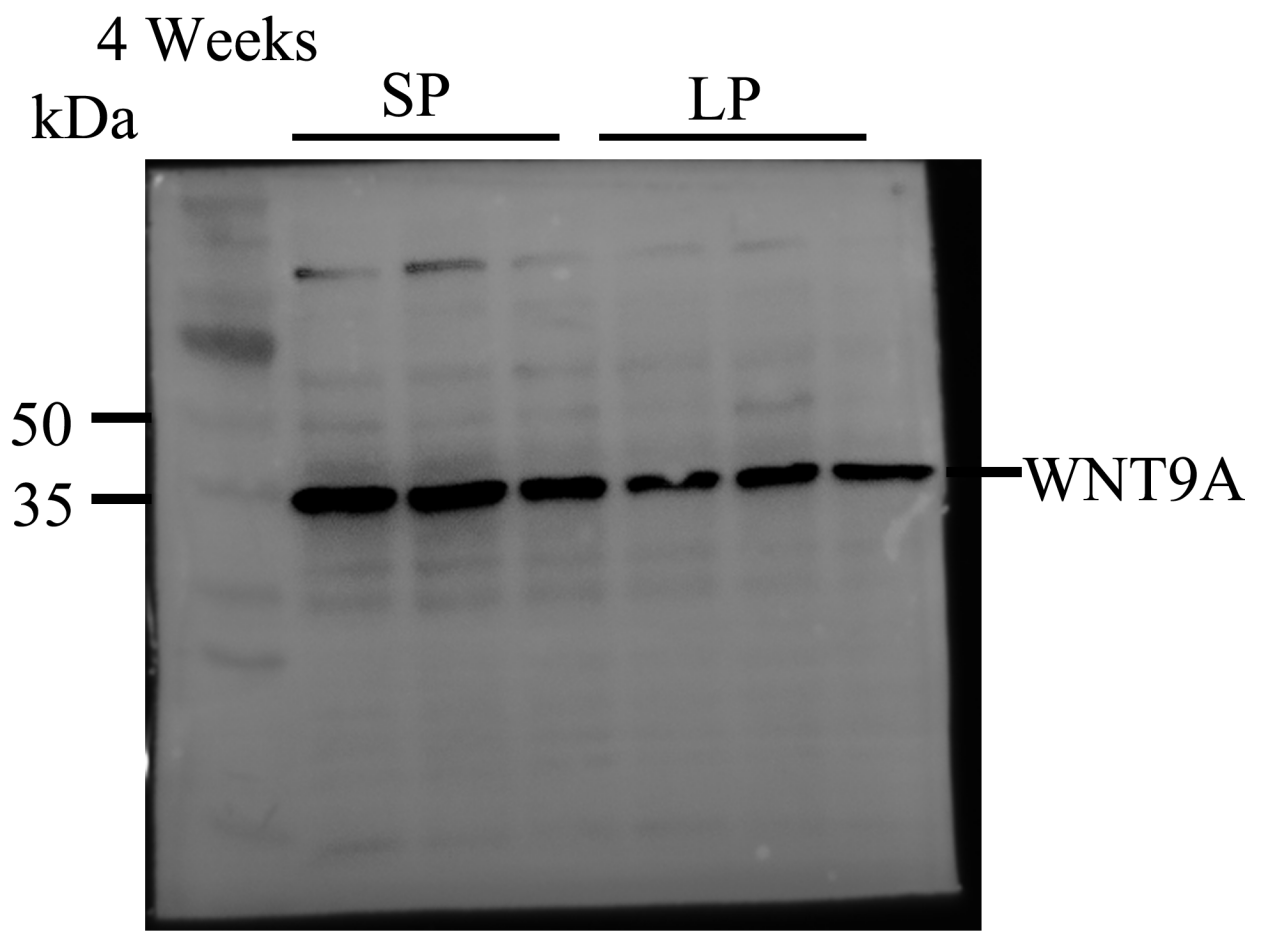

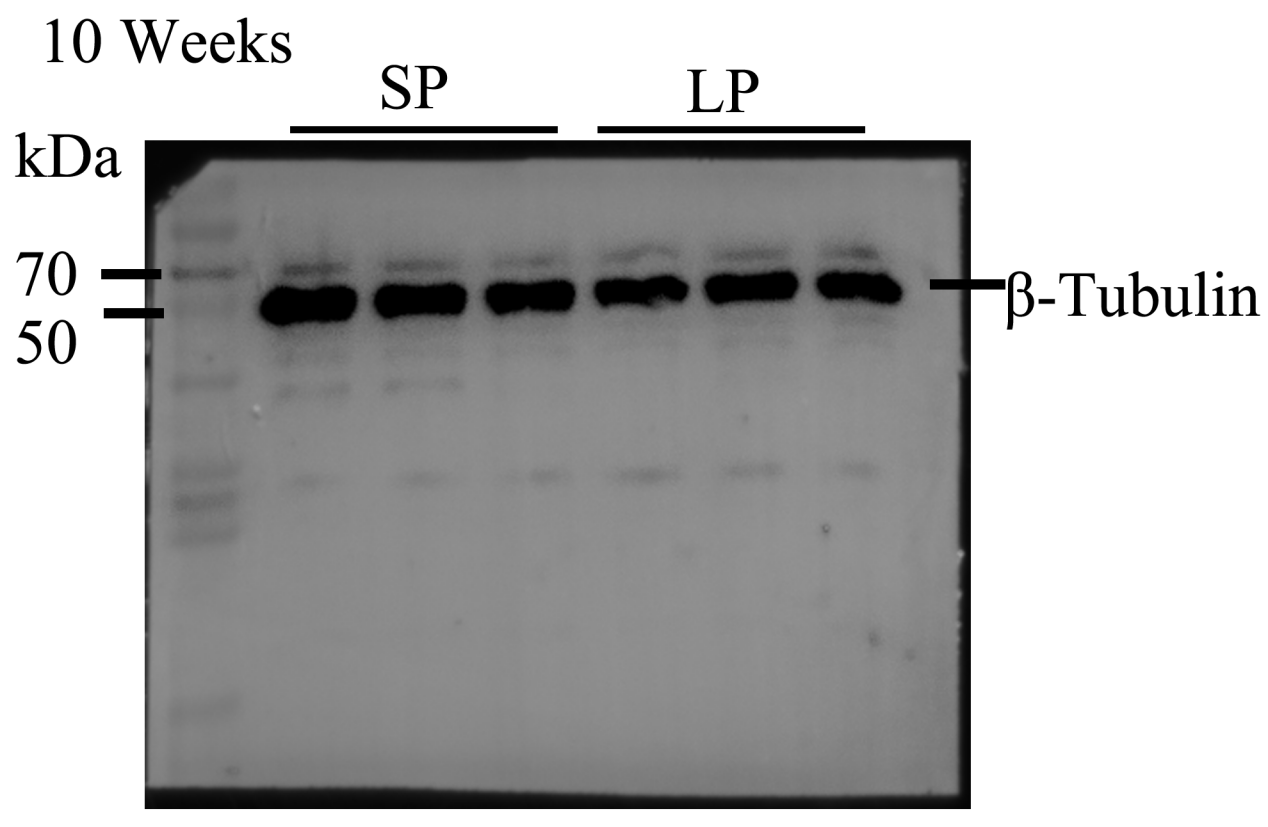

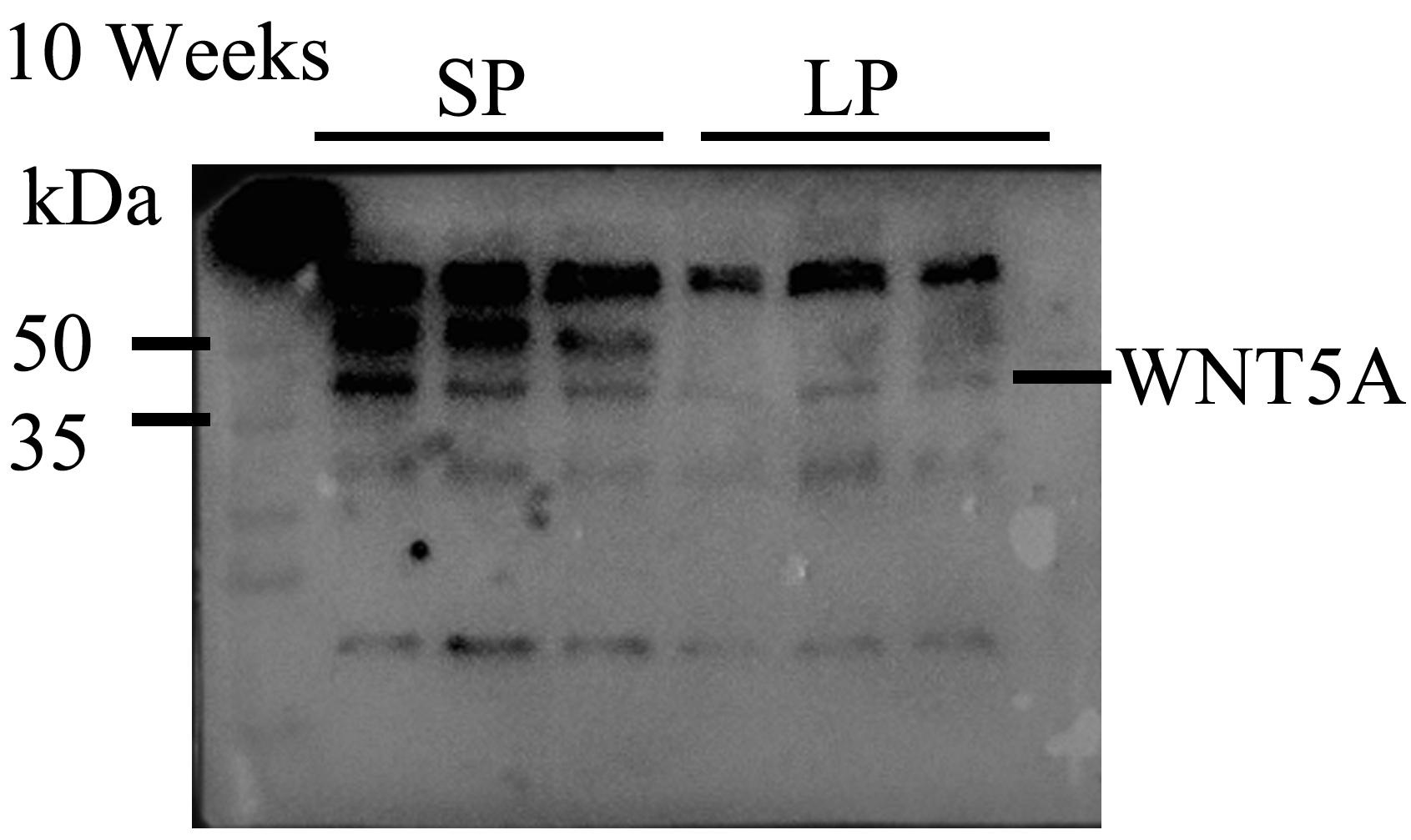

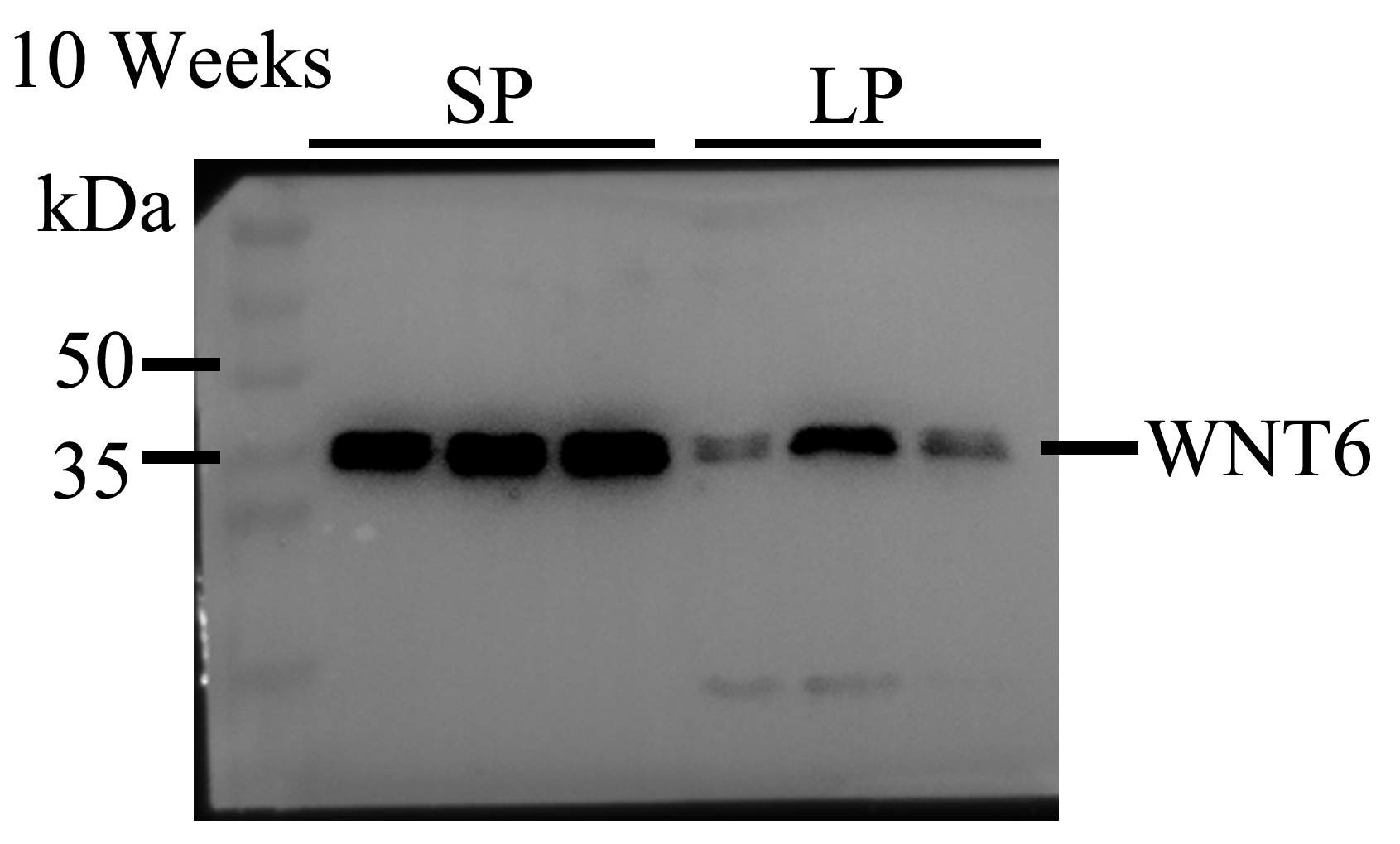

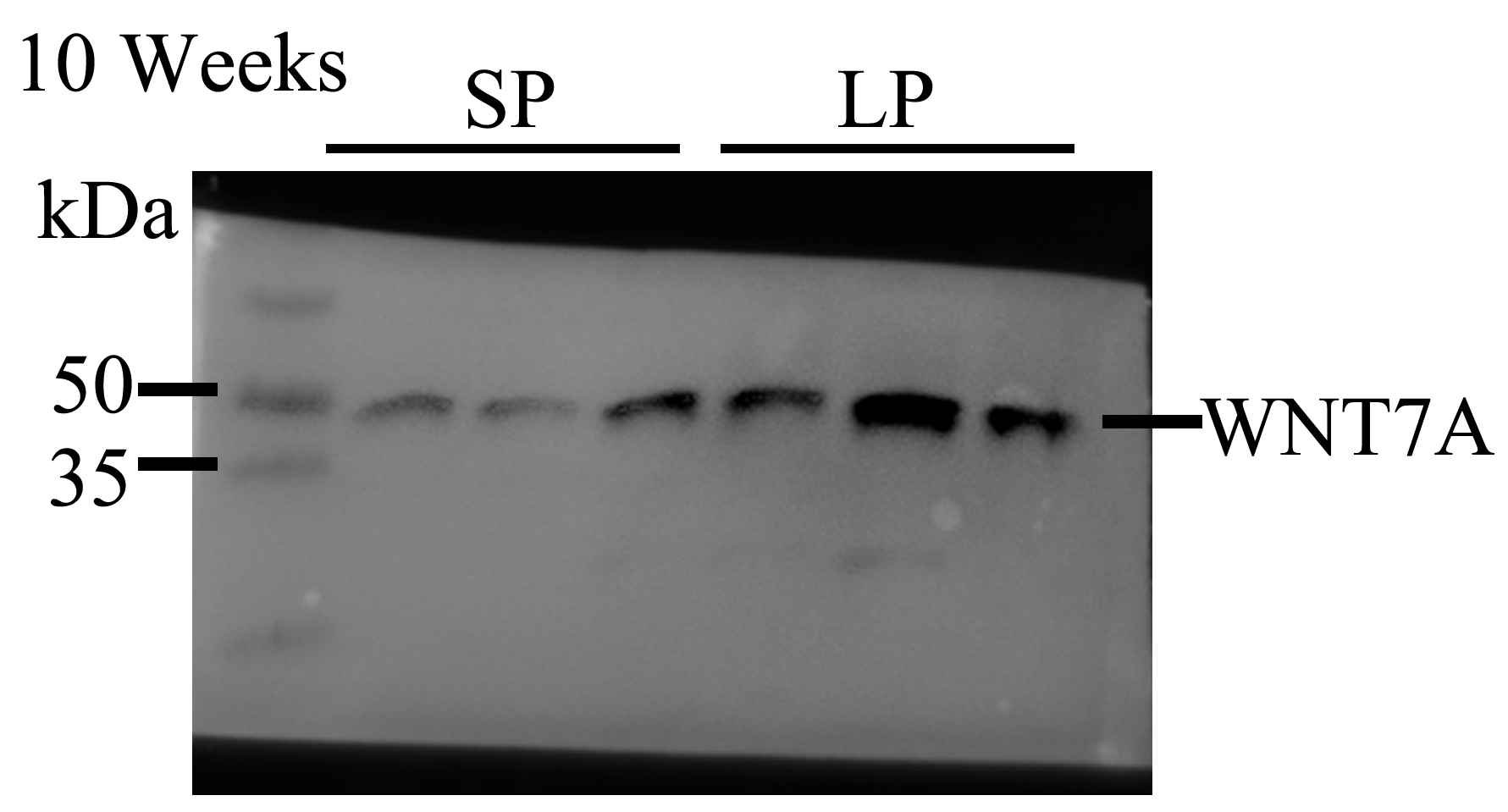

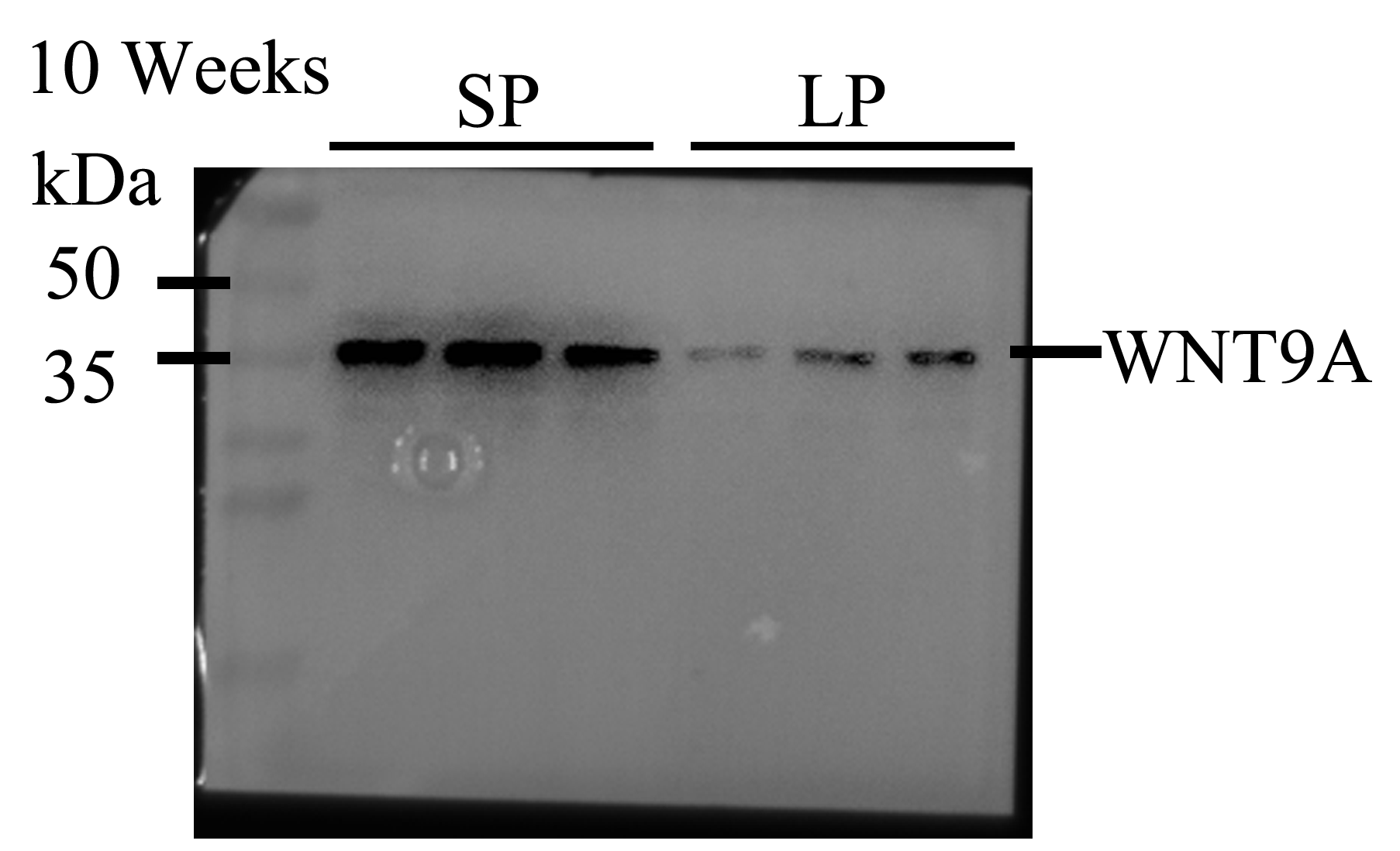

Supplement: Supplementary file 1 — Additional file1 (DOCX 5544 KB) [file 12983_2026_596_MOESM1_ESM.docx]
