# Supplementary material for: Photoperiodic regulation of Wnts in spermatogenesis of Brandt’s vole (Lasiopodomys brandtii)
Source: Front Zool. 2026 Jan 21;23:5. doi: 10.1186/s12983-026-00596-4 (PMC12905888; doi:10.1186/s12983-026-00596-4)
Supplement: Supplementary file 2 — Additional file2 (DOCX 2535 KB) [file 12983_2026_596_MOESM2_ESM.docx]

Supplementary information


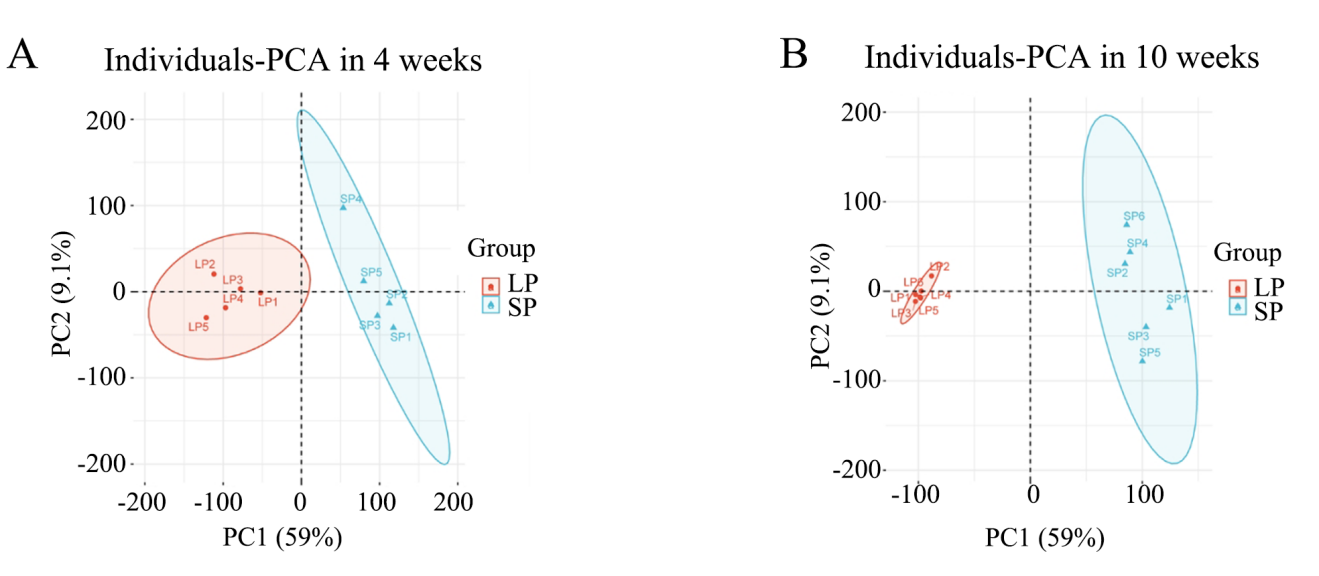


**Supplementary Figure 1.** Principal component analysis (PCA) of testis transcriptomes at 4 (A) and 10 (B) weeks after birth, showing clustering of biological replicates under long and short photoperiods.

**Supplementary Table 1.** Quality control metrics and mapping rates for RNA-seq data from testis samples of Brandt’s voles under LP and SP

| Sample ID | Raw Reads | Clean  Reads | Raw Base(G) | Clean Base(G) | GC content(%) | Q30(%) | Mapping Ratio(%) |
| --- | --- | --- | --- | --- | --- | --- | --- |
| 4LP1 | 26,821,396 | 34,088,225 | 8.05 | 7.65 | 52.05 | 93.64 | 92.71 |
| 4LP2 | 23,175,336 | 25,704,405 | 6.95 | 6.54 | 51.22 | 93.60 | 93.40 |
| 4LP3 | 32,150,710 | 26,443,055 | 9.65 | 9.08 | 50.02 | 93.84 | 92.94 |
| 4LP4 | 24,196,569 | 30,258,034 | 7.26 | 6.83 | 51.36 | 94.97 | 92.75 |
| 4LP5 | 25,704,405 | 27,923,726 | 7.71 | 7.28 | 49.64 | 93.76 | 93.21 |
| 4SP1 | 26,443,055 | 21,169,251 | 7.93 | 7.53 | 49.73 | 94.03 | 92.61 |
| 4SP2 | 27,923,726 | 30,289,203 | 8.38 | 7.92 | 51.48 | 94.81 | 92.09 |
| 4SP3 | 21,169,251 | 28,136,664 | 6.35 | 6.09 | 50.59 | 93.17 | 92.48 |
| 4SP4 | 22,802,190 | 32,150,710 | 6.84 | 6.52 | 51.16 | 93.23 | 92.89 |
| 4SP5 | 28,030,380 | 28,030,380 | 8.41 | 8.04 | 49.66 | 93.78 | 92.72 |
| 10LP1 | 23,455,676 | 20,659,438 | 7.04 | 6.70 | 50.61 | 94.06 | 94.28 |
| 10LP2 | 21,458,216 | 21,706,131 | 6.44 | 6.09 | 51.52 | 93.88 | 93.75 |
| 10LP3 | 34,088,225 | 20,112,333 | 10.23 | 9.55 | 50.09 | 93.64 | 94.08 |
| 10LP4 | 29,816,064 | 33,617,218 | 8.94 | 8.20 | 51.91 | 94.62 | 94.48 |
| 10LP5 | 27,908,867 | 32,200,865 | 8.37 | 7.92 | 52.43 | 94.70 | 94.19 |
| 10LP6 | 30,250,789 | 26,313,139 | 9.08 | 8.27 | 51.84 | 94.81 | 94.50 |
| 10SP1 | 24,008,630 | 24,839,353 | 7.20 | 6.77 | 49.81 | 93.83 | 92.28 |
| 10SP2 | 22,018,935 | 28,333,731 | 6.61 | 6.20 | 51.64 | 94.75 | 92.14 |
| 10SP3 | 23,652,894 | 22,774,761 | 7.10 | 6.66 | 50.87 | 93.84 | 91.68 |
| 10SP4 | 27,242,342 | 25,598,043 | 8.17 | 7.68 | 50.36 | 93.84 | 91.68 |
| 10SP5 | 23,263,851 | 26,345,106 | 6.98 | 6.54 | 51.28 | 94.96 | 92.56 |
| 10SP6 | 21,439,167 | 19,880,787 | 6.43 | 6.03 | 49.62 | 93.82 | 92.27 |

**Supplementary Table 2.** List of 151 genes in the Wnt signaling pathway retrieved from KEGG database for transcriptomic analysis

| Gene | Gene | Gene | Gene | Gene | Gene | Gene |
| --- | --- | --- | --- | --- | --- | --- |
| Apc | Csnk2a2 | Fzd10 | Mapk9 | Prickle4 | Senp2 | Vangl2 |
| Apc2 | Csnk2b | Fzd2 | Myc | Prkaca | Serpinf1 | Wif1 |
| Apcdd1 | Ctbp1 | Fzd3 | Nfatc1 | Prkacb | Sfrp1 | Wnt1 |
| Axin2 | Ctbp2 | Fzd4 | Nfatc2 | Prkca | Sfrp2 | Wnt10a |
| Bambi | Ctnnb1 | Fzd5 | Nfatc3 | Prkcb | Sfrp4 | Wnt11 |
| Btrc | Ctnnd2 | Fzd6 | Nfatc4 | Prkcg | Sfrp5 | Wnt16 |
| Cacybp | Cul1 | Fzd7 | Nkd1 | Psen1 | Skp1 | Wnt2 |
| Camk2a | Daam1 | Fzd8 | Nkd2 | Rac1 | Smad3 | Wnt2b |
| Camk2b | Daam2 | Fzd9 | Nlk | Rac2 | Smad4 | Wnt3a |
| Camk2d | Dkk1 | Gpc4 | Notum | Rac3 | Sost | Wnt4 |
| Camk2g | Dkk2 | Gsk3b | Plcb1 | Rbx1 | Sox17 | Wnt5a |
| Cby1 | Dkk4 | Invs | Plcb2 | Rhoa | Tbl1x | Wnt5b |
| Ccdc88c | Dvl1 | Jun | Plcb3 | Rnf43 | Tbl1xr1 | Wnt6 |
| Ccn4 | Dvl2 | Lef1 | Plcb4 | Rock2 | Tcf7 | Wnt7a |
| Ccnd1 | Dvl3 | Lgr4 | Porcn | Ror1 | Tcf7l1 | Wnt7b |
| Ccnd2 | Ep300 | Lgr5 | Ppard | Ror2 | Tcf7l2 | Wnt8b |
| Ccnd3 | Fbxw11 | Lgr6 | Ppp3ca | Rspo1 | Tle1 | Wnt9a |
| Cer1 | Fosl1 | Lrp5 | Ppp3cb | Rspo2 | Tle2 | Wnt9b |
| Chd8 | Frat1 | Lrp6 | Ppp3r1 | Rspo3 | Tle3 | Znrf3 |
| Crebbp | Frat2 | Map3k7 | Prickle1 | Rspo4 | Tle4 |  |
| Csnk1e | Frzb | Mapk10 | Prickle2 | Ruvbl1 | Tle6 |  |
| Csnk2a1 | Fzd1 | Mapk8 | Prickle3 | Ryk | Vangl1 |  |


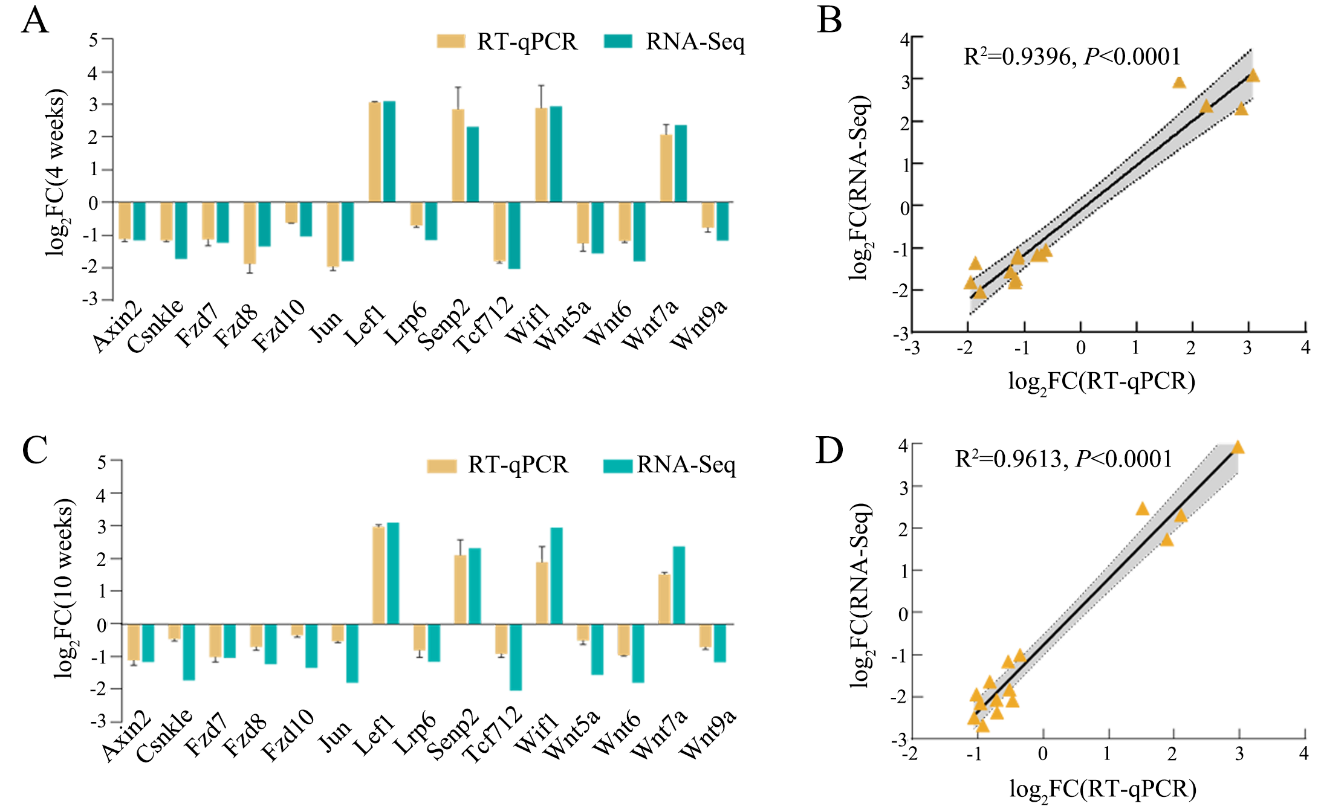


**Supplementary Figure 2.** Correlation analysis between RNA-Seq and RT-qPCR. Bar chart of fold change (A) and scatter diagram of correlation (B) between RNA-Seq and RT-qPCR at 4 weeks after birth. Bar chart of fold change (C) and scatter diagram of correlation (D) between RNA-Seq and RT-qPCR at 10 weeks after birth.
